# Factors associated with the use of deworming drugs during pregnancy in Tanzania; an analysis from the 2015–16 Tanzanian HIV and malaria indicators survey

**DOI:** 10.1186/s12884-021-04291-6

**Published:** 2022-01-22

**Authors:** Vicent Bankanie, Fabiola Vincent Moshi

**Affiliations:** 1 Department of Clinical Nursing, School of Nursing, the University of Dodoma, P.O. Box 259, Dodoma, Tanzania; 2 Department of Nursing Management and Education, School of Nursing, the University of Dodoma, P.O. Box 259, Dodoma, Tanzania

**Keywords:** Pregnancy, Helminths, Parasite, Deworming drugs, Mebendazole, Anaemia

## Abstract

**Background:**

The use of deworming drugs is one of the important antenatal strategies in preventing anaemia in pregnancy. Little is known about the factors associated with the use of deworming drugs, which accounts for the aim of this study.

**Method:**

The study used data from the 2015–16 Tanzania HIV Demographic and Health Survey and Malaria Indicators Survey (2015–16 TDHS-MIS). A total of 6924 women of active reproductive age from 15 to 49 were included in the analysis. Both univariate and multiple logistic regression analyses were used.

**Results:**

The majority of interviewed women 3864(60.1%) took deworming drugs. In a weighed multiple logistic regression, women residing in urban areas reported greater use of deworming drugs than women residing in rural areas [AOR = 1.73, *p* = 0.01, 95% CI (1.26–2.38)]. In the four areas of residence, compared to women residing in mainland rural areas, women residing in mainland urban areas and Pemba islands reported greater use of deworming drugs [mainland urban (AOR = 2.56 *p* < 0.001,95% CI(1.78–3.75), and Pemba Island (AOR = 1.18, *p* < 0.001, 95% CI(1.17–1.20)]. However, women residing in Zanzibar Island (Unguja) were less likely to use deworming drugs compared to women in mainland rural women (AOR = 0.5, *p* < 0.001, 95% CI (0.45–0.55).

Similarly, compared to women under 20 years of age, women between 20 to 34 years reported significantly greater use of deworming drugs [20 to 34 years (AOR = 1.30, *p* = 0.03, 95% CI(1.02–1.65).

Likewise, greater use of deworming drugs was reported in women with a higher level of education compared to no education [higher education level (AOR = 3.25, *p* = 0.01,95% CI(1.94–7.92)], rich women compared to poor [rich (AOR = 1.43, *p* = 0.003, 95% CI (1.13–1.80)] and in women who initiated antenatal care on their first trimester of pregnancy compared to those who initiated later [AOR = 1.37, *p* < 0.001, 95% CI (1.17–1.61)].

**Conclusion:**

Women who were more likely to use the deworming drugs were those residing in urban compared to rural areas, aged between 20 and 34 years, those with a higher level of formal education, wealthier, and women who book the antenatal clinic (ANC) within their first trimester of pregnancy. Considering the outcomes of anaemia in pregnancy, a well-directed effort is needed to improve the use of deworming drugs.

## Background

Deworming drugs are usually given to pregnant women in endemic areas to treat possible helminthiasis infection. Helminthiasis is a macro parasitic disease in which a part of the human body, mainly the intestine, is infected with parasitic worms, known as helminths. About a quarter of the general world population [1], and more than one-third of all pregnant women in developing countries are infected with one or more of the intestinal helminths. The prevalence varies with geographical locations. In some endemic regions, the prevalence of helminths in pregnant women is as high as 70% [2].

People can be infected by ingestion of viable eggs through food or water (*A. lumbricoides* and *T. trichiura*), or by skin penetration when walking barefooted on contaminated soil (Hookworm). *Ancylostoma duodenale,* (a species of hookworm) can also be transmitted through the ingestion of larvae. Inside the human body, the adult parasite produces eggs, which are passed in human faeces and deposited in the external environment on defecation, where they can stay viable for several months for re-infection. The adult helminths cause internal bleeding, intestinal obstruction and inflammation, the release of anticoagulant substances, and reduced availability of nutrients necessary for the erythropoiesis process through anorexia, vomiting, and diarrhoea [3]. All these contribute to iron deficiency anaemia. In Tanzania, iron-deficiency anaemia in pregnancy due to helminthic infection contributes to increased anaemia-related maternal and neonatal mortality, premature delivery, and low birth weight, especially in endemic regions [4].

Helminthic infection among pregnant women is related to several factors. A recent study in Ethiopia revealed an association between elevated helminthiasis infection and illiteracy, absence of latrine, and regular consumption of raw and/or unwashed fruit among pregnant women [5]. Therefore, while administration of deworming drugs during antenatal visits remains important, health education on hygiene and wearing shoes can also help to prevent helminth infections.

Despite a few inconsistencies in some studies regarding the antenatal effect of deworming [6], WHO recommends preventive chemotherapy using single-dose albendazole (400 mg) or mebendazole (500 mg) in pregnant women in areas where the prevalence of anaemia in pregnancy is 40% or higher and the baseline prevalence of hookworm and/or T, trichiura infection is 20% or higher [7]. In a randomised controlled trial to compare the pregnancy outcomes in Nigeria among those who received 500 mg mebendazole and those who did not, the prevalence of anaemia at 37 weeks gestation and above in the treatment arm was significantly lower than in the placebo group (12.6% vs 29.9%, *p* < 0.001) [8]. Similar findings have been reported elsewhere [9].

In several endemic regions, especially sub-Saharan Africa, the control of hookworm and other geohelminth infections is incorporated in routine antenatal care programmes. Therefore, when women attend antenatal clinics (ANC), they freely obtain the drugs and take them in as a Directly Observed Treatment (DOT) to improve adherence. However, as reported in other studies regarding drug compliance in pregnancy [10, 11], some pregnant women may avoid the drugs due to uncomfortable side effects such as nausea, vomiting, and diarrhoea, associated with chewable mebendazole tablets [12], especially when given with other routine drugs such as antimalarials (Sulphadoxine Pyrimethamine) for malaria prevention. Therefore, in most ANC situations where monitoring is difficult due to increased workload among ANC care providers, adherence may remain a challenge despite the DOT strategy. In Tanzania, when the drugs are out of stock [13], pregnant women are instructed to buy and use the drugs in the absence of healthcare providers which also increases the chance for non-adherence to the drugs.

In Tanzania, deworming by mebendazole 500 mg for all pregnant women attending ANC across the country remains routine in ANC [14]. However, while preconception anaemia remains a challenge in Tanzania [15], and the overall prevalence of anaemia among women of reproductive age increased from 40% in 2010 to 45% in 2015–16 [14], it remains higher among pregnant women (57%) compared to both breastfeeding mothers (46%) and women who are neither pregnant nor breastfeeding (43%) [14]. Among several other questions regarding this increased proportion of anaemia, is whether they truly use the deworming drugs given during their antenatal visits and the associated factors for the use. Therefore, this retrospective study was conducted to discover factors associated with the use of deworming drugs during pregnancy among women of reproductive age in Tanzania.

## Methods

### Study design and data

This study was a cross-sectional analysis of a dataset from the 2015–16 Tanzania Demographic and Health Survey and Malaria Indicator Survey (TDHS-MIS) dataset (2015-16TDHS-MIS). The dependent variable was the use of deworming drugs. The independent variables were: 1) Antenatal booking (early compared to late antenatal booking): In the TDHS-MIS, early antenatal booking refers to having the first antenatal visit during the first trimester of pregnancy, while late antenatal booking refers to the first antenatal visit after the first trimester of pregnancy [14].

2) age group of a woman during the TDHS-MIS (20–34, and more than 34, compared to age less than 20 years), 3) place of residence (urban compared to rural), 4), area of residence (mainland urban, Unguja and Pemba compared to mainland urban), 5) wealth index (middle and rich compared to poor), 6) parity (para 2–4, and para 5+ compared to para 1), and 7) level of education (primary, secondary and higher education compared to no education).

The TDHS-MIS also collected information about household wealth, and categorized the participants into groups by principal component analysis [14]. Households were given scores based on the number and kinds of consumer goods they own, ranging from a television to a bicycle or car, plus housing characteristics, such as the source of drinking water, toilet facilities, and flooring materials. National wealth quintiles were compiled by assigning the household score to each usual (de jure) household member, ranking each person in the household population by their score, and then dividing the distribution into five equal categories, each with 20% of the population [14].

### The 2015–16 TDHS-MIS

The details about the 2015–16 TDHS-MIS has been described in the respective DHS report [14]. Brieflly, the 2015–16 TDHS-MIS is the sixth in series of the Tanzania Demographic and Health Survey and Malaria Indicator Survey, with the primary objective of providing up-to-date estimates of basic demographic and health indicators for successful policy planning and implementation.

The sampling procedure involved two stages to obtain a sample for urban and rural areas in both Tanzania mainland and Zanzibar. In the first stage, a total of 608 sample points (clusters) were identified. These clusters were the enumeration areas (EAs) delineated for the 2012 Tanzania Population and Housing Census [16]. The second stage involved a systematic selection of households, whereby 22 households were selected from each cluster to yield a representative probability sample of 13,376 households. A more detailed description of the sampling technique has been described in the TDHS-MIS report [14].

To enhance representativeness Tanzania was divided into nine geographic zones namely Western Northern zone, Central zone, Southern Highland zone, Southern zone, South-West Highland zone, Lake zone, Eastern zone, and Zanzibar and a representative sample obtained from each of the zones.

To ensure statistical representation of the whole country, the distribution of the women in the sample was weighted (or mathematically adjusted) such that it resembles the true distribution in the country. Oversampled women from a small region contributed a small amount to the national total while undersampled women from a large region, like Dar es Salaam, contributed much more. In this way, the “weight” was calculated, which was used to adjust the number of women from each region so that each region’s contribution to the total is proportional to the actual population of the region [14].

Data collection involved four questionnaires based on the DHS program’s standard and have been described and published in the DHS report [14].

In this study, the data analysis process used data obtained from the Woman’s Questionnaire containing information from all eligible women aged 15–49 years. The information collected includes background characteristics, birth history and childhood mortality, knowledge and use of family planning methods, fertility preferences, access to antenatal services, delivery, and postnatal care, breastfeeding and infant feeding practices, vaccinations and childhood illnesses, marriage and sexual activity, women’s work and husbands’ background characteristics, adult mortality, including maternal mortality, malaria, domestic violence, and other health-related issues.

### Study population and data extraction

The study population included all women of reproductive age (aged 15–49 years). The study used Individual file recode (TZIR7BFL). The analysis included only women who remembered the timing for antenatal booking of their youngest child and /or had an antenatal card for the most recent pregnancy for reference [14]. Those who had not been able to recall the timing (which included those who had no antenatal card for reference) or those who did not respond to the question on whether the woman took any drug for intestinal worms were removed from the analysis.

### Statistical analysis

Statistical Package for Social Sciences (IBM SPSS version 20) was employed for data analysis. Both weighed and unweighed data analysis were performed. A weighted data analysis was done by using weights determined by the DHS statisticians in order to increase the representativeness of the sample [14]. The variables which were presumed to influence the use of deworming drugs during pregnancy were filtered from the DHS-MIS and were first described in terms of percentage and frequencies. Then, the association between the dependent and independent variables was assessed by using the Chi-squared test. The variables that revealed a significant association were fitted in a binary weighted logistic regression model independently and the crude odds ratios (COR) established. Afterwards, all variables were entered into the model and adjusted odds ratios (AOR) were established to determine the significant independent predictors of the use of deworming drugs. Variables were considered to be significantly associated with the use of deworming drugs if *p*-value< 5%.

## Results

### Socio-demographic characteristics

The study used individual file recode (TZIR7BFL) which had a total of 13,266 women who responded to the survey (97% response rate). The weighed and unweighed analysis revealed no statistical significant differences. Therefore, the weighed analysis results were reported. A total of 6924 women of reproductive age who had given birth within 5 years preceding the survey, remembered the timing of the first antenatal visit for their last child. The majority of study respondents 5113 (73.8%) resided in the rural setting of Tanzania, were aged 20 to 34 years 4557(65.8%), had only primary education 4209 (60.8) and were married 5650 (86.1%) (Table 1).

**Table 1:** Socio-demographic characteristics of women who knew their antihelminthic drug status during their most recent pregnancy^a^, Tanzania DHS^b^, 2015–6

Variables	Frequency	Per cent (%)
**Place of residence**		
Rural	5113	73.8
Urban	1811	26.2
**Area of residence (Mainland/Zanzibar)**		
Mainland rural	4357	62.9
Mainland urban	1618	23.4
Unguja (Zanzibar Island)	594	8.6
Pemba (Pemba Island)	355	5.1
**Age group of a woman (At the time of TDHS**^**b**^**-MIS)**		
Less than 20 years	541	7.8
20 to 34 years	4557	65.8
More than 34 years	1826	26.4
**Educational level**		
No education	1329	19.2
Primary education	4209	60.8
Secondary	1326	19.2
Higher	60	0.9
**Respondent currently working**		
Not working for money	1498	21.6
Working for money	5426	78.4
**Wealth index**^**c**^		
Poor	2734	39.5
Middle	1363	19.7
Rich	2827	40.8
**Marital Status**		
Never in union	441	6.4
Married	5650	81.6.
Widow	119	1.7
Separated	714	10.3
**Parity**		
Para one	1595	23
Para 2–4	3154	45.6
Para 5+	2175	31.4
**Antenatal booking**^**d**^		
Early booking	1586	22.9
Late booking	5338	77.1
**Number of antenatal visits**		
Less than 4 visits	3438	49.7
Four 4 or more visits	3486	50.3

^a^Ages 15–49 at the time of the survey who had a pregnancy in the previous 5 years

^b^TDHS-MIS-Tanzania Demographic and Health Survey and Malaria Indicator Survey

^c^Wealth quintiles were determined for the DHS based on using principal components analysis on a list of possessions and housing characteristics

^d^Early booking means first antenatal visit during the first trimester, while late booking means first antenatal visit after the first trimester of pregnancy

### The relationship between women’s characteristics and use of deworming drugs in Tanzania

The association between the dependent and independent variables was assessed by using the Chi-squared test. Women’s characteristics that showed a significant relationship with the use of deworming drugs were the timing of antenatal booking (*p* < 0.001), place of residence (*p* < 0.001), age group (*p* < 0.001), an education level (*p* < 0.001), parity (*p* < 0.001), wealth index (*p* < 0.001), Zones of residence (*p* < 0.001) and the number of antenatal visits (*p* < 0.001).

### Factors associated with the use of deworming drugs during pregnancy in Tanzania

After controlling for confounders (other independent variables), in a weighed multiple logistic regression, women residing in urban areas reported greater use of deworming drugs than women residing in rural areas [AOR = 1.73, *p* = 0.01, 95% CI (1.26–2.38)]. In the four areas of residence, compared to women residing in mainland rural areas, women residing in mainland urban areas and Pemba islands reported greater use of deworming drugs [mainland urban (AOR = 2.56 *p* < 0.001,95% CI(1.78–3.75), and Pemba Island (AOR = 1.18, *p* < 0.001, 95% CI(1.17–1.20)]. However, women residing in Zanzibar Island (Unguja) were less likely to use deworming drugs compared to women in mainland rural areas (AOR = 0.5, *p* < 0.001, 95% CI (0.45–0.55).

Similarly, compared to women under 20 years of age, women between 20 to 34 years reported significantly greater use of deworming drugs [20 to 34 years (AOR = 1.30, *p* = 0.03, 95% CI(1.02–1.65).

Likewise, greater use of deworming drugs was reported in women with a higher level of education compared to no education [higher education level (AOR = 3.25, *p* = 0.01,95% CI(1.94–7.92)], rich women compared to poor [rich (AOR = 1.43, *p* = 0.003, 95% CI (1.13–1.80)] and in women who initiated antenatal care on their first trimester of pregnancy compared to those who initiated later (in the second and third trimesters) [AOR = 1.37, *p* < 0.001, 95% CI (1.17–1.61)].

## Discussion

In the present study, the proportion of women who reported using deworming drugs during their most recent pregnancy was 60.1%. This proportion is lower than the proportion of women who attend at least one antenatal visit in Tanzania, which is about 98% [14]. This implies that a significant proportion of pregnant women attend ANC but do not use deworming drugs. Although there is limited information regarding the reasons for the nonuse of deworming drugs in Tanzania, the lower proportion of pregnant women using deworming drugs may be related to the drug unavailability (Out-of-stock), women’s lack of knowledge regarding the use of deworming drugs during pregnancy, and increased workload among antenatal healthcare providers, which may produce clinician mistakes and inadequate supervision during DOT.

In Tanzania, like other Low and Mid-Income Countries (LMICs), the budget invested for health expenditure is low. In such countries, the proportion of total health expenditure spent on medicines ranges widely from 7.7 to 67.6% [13], which may account for the occasional lack of some important drugs in health facilities. Likewise, in Tanzania, the out-of-stock may result from problems in the supply chain of ordered drugs, lack of priority in ordering drugs, and lack of measures for holding accountable those who were responsible for the lack of drugs [17]. In a recent study in Dodoma, Tanzania, Mebendazole was available in 72.7% of health centres and 73.1% of dispensaries in the past 3 months prior to the survey [13].

In these situations of limited availability of drugs, clients are usually advised to buy and use the deworming drugs on their own without clinician supervision. In subsequent visits, the women may falsely report to clinicians to have used the drug at home.

Furthermore, most ANCs in Tanzania operate under staff shortage, which may subject clinicians to several errors including incorrect documentation that the drug was given, or failure to monitor closely whether the woman used the drug provided to her during DOT. While some pregnant women may refuse to receive the drug, other women may receive but pretend to chew the mebendazole tablets to avoid the associated side effects such as nausea, vomiting, and diarrhoea that may complicate the pre-existing complications of pregnancy.

In this study, the factors associated with the greater use of deworming drugs include early antenatal booking, residing in urban compared to rural, Zanzibar and Pemba islands, age group between 20 to 34, higher level of formal education, and being wealthier.

Women who book early in the ANC are more likely to use the deworming drugs than those who book later. Studies in Ethiopia revealed a relationship between early booking in the ANC and higher knowledge regarding the use of antenatal services [18]. Therefore, those who book early in the ANCs may be more knowledgeable about the importance of using deworming drugs during pregnancy than those who book later or never. These findings underscore the importance of reproductive health education including the use of deworming drugs among women of reproductive age even before they become pregnant, and whenever possible, beyond ANCs. Furthermore, women who book early are likely to have more antenatal visits. Therefore, the woman is likely to get the drugs in her subsequent visits if stock out was an issue, or if she was mistakenly deprived in her previous antenatal visit. However, in the 2015–16 TDHS-MIS report, only 24% of women started ANC at their first trimester of pregnancy [14].

Regarding the influence of geographical residence on the use of deworming drugs, (Table 2), women in urban areas are more likely to use deworming drugs than women in rural areas [AOR = 1.73, *p* = 0.01, 95% CI (1.26–2.38)]. Similarly, compared to women residing in mainland rural areas, women residing in mainland urban areas and Pemba islands reported greater use of deworming drugs. In the 2015–16 TDHS-MIS report [14], urban women were more likely than rural women to have four or more ANC visits and to seek care early in pregnancy. In addition, rural women in Tanzania report a higher rate of delayed ANC initiation and illiteracy rate compared to urban women [14], similar to the findings in Ethiopia [5, 19]. All these factors suggest an increased rate of use of deworming drugs in urban compared to rural areas.

**Table 2:** Factors associated with the use of deworming drugs among women during their most recent pregnancy^a^, Tanzania DHS-MIS^b^, 2015–16

Variable	OR^c^	95%CI		*P*-value	AOR^d^	95%CI		*P*-value
		Lower	Upper			Lower	Upper	
**Place of residence**								
Rural	1				1			
Urban	2.00	1.69	2.38	< 0.001	1.73	1.26	2.38	0.01
**Area of residence**								
Mainland rural	1				1			
Mainland urban	2.08	1.75	2.50	< 0.001	2.56	1.78	3.75	< 0.001
Unguja (Zanzibar Island)	0.61	0.57	0.66	< 0.001	0.50	0.45	0.55	< 0.001
Pemba (Pemba Island)	1.24	1.13	1.35	< 0.001	1.18	1.17	1.20	< 0.001
**The age group of a woman (At the time of TDHS-MIS)**								
Less than 20 years	1				1			
20 to 34 years	1.53	1.24	1.89	< 0.001	1.30	1.02	1.65	0.033
More than 34 years	1.26	1.005	1.58	0.046	1.17	0.87	1.57	0.286
**Educational level**								
No education	1				1			
Primary education	1.22	1.06	1.42	0.007	1.06	0.91	1.23	0.47
Secondary	1.73	1.42	2.11	< 0.001	1.24	0.98	1.57	0.07
Higher	5.99	2.39	15.00	< 0.001	3.25	1.94	7.92	0.01
**Wealth index**^**e**^								
Poor	1				1			
Middle	1.20	1.01	1.44	0.04	1.15	0.96	1.38	0.14
Rich	1.99	1.69	2.34	< 0.001	1.43	1.13	1.80	< 0.003
**Parity**								
Para one	1				1			
Para 2–4	1.13	0.97	1.31	0.13	1.14	0.96	1.35	0.13
Para 5+	0.84	0.71	0.98	0.03	1.08	0.85	1.36	0.54
**ANC Booking**^**f**^								
Late booking	1				1			
Early booking	1.62	1.38	1.89	< 0.001	1.37	1.17	1.61	< 0.001
**Number of antenatal visits**								
Less than 4 visits	1				1			
Four or more visits	1.41	1.24	1.60	< 0.001	1.13	0.99	1.28	0.080

^a^Ages 15–49 at the time of the survey who had a pregnancy in the previous 5 years

^b^TDHS-MIS**-**Tanzania Demographic and Health Survey and Malaria Indicator Survey

^c^Odd Ratio

^d^Adjusted Odd Ratio

^e^Wealth quintiles were determined for the DHS based on using principal components analysis on a list of possessions and housing characteristics

^f^Early booking means first antenatal visit during the first trimester, while late booking means first antenatal visit after the first trimester of pregnancy

In this study, women between 20 and 34 years of age were more likely to use deworming drugs than women below 20 years. These findings may have multiple explanations: In Tanzania, the majority of teenage pregnancies come from poor families [14] which is a significant factor for poor use of deworming drugs and late booking to ANCs. Poor women are least likely to book early and/or to have multiple ANC visits, which lowers their chance to receive drugs from ANC when stock out is a challenge. Similarly, when drugs are out of stock, poor women are least likely to afford the deworming drugs.

Secondly, considering that the median age for marriage among women in Tanzania is 19.2 years [14], then the majority of pregnant women below 20 years are likely to be primigravida and/or unmarried. Primigravida women have little antenatal experience and are therefore likely to have inadequate knowledge regarding ANC services and the use of deworming drugs compared to multigravida/multipara women who have had one or more previous antenatal experiences. Lack of knowledge means the women may not realize the importance of using the deworming drugs and the dangers associated with defaulting in case of stock-out. In a recent study in Sri Lanka, multiparous women (2–4 children) were more knowledgeable about complication readiness in pregnancy than nulliparous women [20]. Similarly, our study reveals greater use of deworming drugs in multiparous than nulliparous women. In our study there was very little effect of parity when controlling for age (Table 2). Likewise, being unmarried is associated with unplanned pregnancy [21]. Unlike women with unplanned pregnancies [22], the women with planned pregnancies are likely to have early initiation of antenatal services and/or multiple ANC attendance [18], and health-seeking behaviour [23] which are important factors for use of deworming drugs.

In this study, women with a higher level of formal education (beyond secondary school) were more likely to use the deworming drugs than women without formal education [(AOR = 3.25, *p* = 0.02,95% CI(1.94–7.92)]. More intriguingly, recent evidence reveals that the odds of intestinal parasitic infection among illiterate pregnant mothers are more than 2-fold higher compared to the educated pregnant mothers [2]. Combining the two observations, it can be suggested that, women with formal education are likely to be more knowledgeable on the importance of hygiene, compliance to deworming drugs, dangers of defaulting, and health-seeking behaviour than those without formal education. Besides, formal education by pregnant women is associated with early ANC initiation [19], an important factor for the use of deworming drugs. Furthermore, richer and more educated women are more able to act on their knowledge than less educated women. With these findings, the national and international call for women’s educational empowerment would improve the use of deworming drugs and other antenatal services, among several other benefits to women.

We also found that wealthier women are more likely to use deworming drugs than the poor [rich (AOR = 1.43, *p* < 0.001, 95% CI (1.13–1.80)]. This has a dual explanation: First, being wealthier implies that women are, not only able to afford transport to the clinic where they can access the services, but also able to afford the deworming drugs from the pharmaceutical stores when the ANC clinics run out of stock [22]. In such situations, only those with the ability to afford the drugs are likely to use the drug. The influence of wealth on the utilization of ANC has been reported in Nigeria [24], and Ghana [25]**.** Second, wealthier and employed women are more likely to book ANCs early than unemployed [24], which is a significant factor for the use of deworming drugs. This accounts for the importance of women’s economic empowerment in improving ANC utilization including use of the deworming drugs.

### Strength and limitation of the study

The strength of this study is that it was community-based which covered participants from all the nine zones of Tanzania. Therefore, the findings may represent the factors associated with the use of deworming drugs during pregnancy among women of reproductive age in Tanzania. However, being a retrospective study, it included only women who remembered the timing for antenatal booking of their youngest child, which may lead to recall bias.

## Conclusion

The use of deworming drugs is lower than the expected proportion of women attending ANC in Tanzania, which is 98%. Women who were more likely to use deworming drugs were those residing in urban areas, attend the ANCs within their first trimester of pregnancy (early antenatal booking), age above 20 years, with a higher level of formal education and wealthier women. Considering the outcomes of anaemia in pregnancy, a well-directed effort is needed to improve the use of deworming drugs. Such effort may include educating women in the groups with the lowest use of deworming drugs of the importance of using the deworming drugs during pregnancy, and the dangers associated with defaulting. Whenever possible, it should be done even beyond ANCs.

Given the limited supply of deworming drugs, the ANCs should be kept stocked, especially in the poor, rural areas, and in clinics where most women have a low level of education. When the drugs are out of stock, and the pregnant women required to buy on their own, emphasis should be provided on the importance of use and the risks associated with skipping the drugs. Furthermore, the national and international call for women educational and economic empowerment should be supported for successful achievement of health interventions to improve women health, including the use of deworming drugs and the utilization of other antenatal services.

## Data Availability

The data that support this analysis are available from the 2015–16 Tanzania HIV and Malaria Indicators Survey (THMIS). Data is available from the authors upon reasonable request and with permission from MEASURE DHS.

## Abbreviations

ANC: Antenatal Clinic
TDHS: Tanzania Demographic Health Survey
TDHS-MIS: Tanzania Demographic Health Survey and Malaria Indicator Survey
DOT: Directly Observed Treatment
AOR: Adjusted Odd Ratio
